# Understanding multicaloric effects in anisotropic magnets via a mean-field approach

**DOI:** 10.1080/14686996.2025.2517528

**Published:** 2025-06-11

**Authors:** Yulia Klunnikova, Alex Y. Karpenkov, Benedikt Beckmann, Wei Liu, Konstantin P. Skokov

**Affiliations:** Institute of Materials Science, Technical University of Darmstadt, Darmstadt, Germany

**Keywords:** Magnetocaloric effect, magnetic anisotropy, mean-field approach

## Abstract

Materials with magnetic anisotropy can serve as a model object for exploring the multicaloric effect because their thermodynamic state alterations can be achieved either through the application of a magnetic field *H* or/and by mechanically rotating the sample in the magnetic field using torque *τ*. In such materials, the total entropy change $\Delta S_{T}$ arises from two distinct contributions: (1) the conventional magnetocaloric effect (MCE) or paraprocess $\Delta S_{\left|m\right|}$ and (2) the rotational MCE $\Delta S_{\varphi }$. In this manuscript, using molecular field model which enables a separation of contributions to the total entropy change $\Delta S_{T}$ from conventional $\Delta S_{\left|m\right|}$ and rotational $\Delta S_{\varphi }$, we have determined cross-coupling multicaloric coefficients $\chi_{\tau ,H}={\left(\frac{\partial \tau }{\mathrm{\partial H}}\right)}_{T,\theta }$ and $\chi_{H,\tau }=-{\left(\frac{\mathrm{\partial m}}{\partial \theta }\right)}_{T,H}$ for anisotropic magnetic materials and show that they satisfy the basic thermodynamic identities. We also confirmed that the total multicaloric effect in the material with magnetic anisotropy can be accurately expressed as the sum of the individual magnetocaloric effects induced by separate application of the *H* and *τ*, minus the magnetic entropy change arising from thermodynamic cross-coupling between the subsystems of the solid: $\;\Delta S_{T}=\Delta S_{T,\tau }^{\left(H\right)}+\Delta S_{T,H}^{\left(\theta \right)}-\;\Delta S_{\mathrm{coupling}}$.

## IMPACT STATEMENT

This work first treats an anisotropic magnet as a multicaloric material, calculating cross-coupling coefficients via mean-field theory and determining the cross-coupling magnetic entropy change.

## GRAPHICAL ABSTRACT

**Figure:**
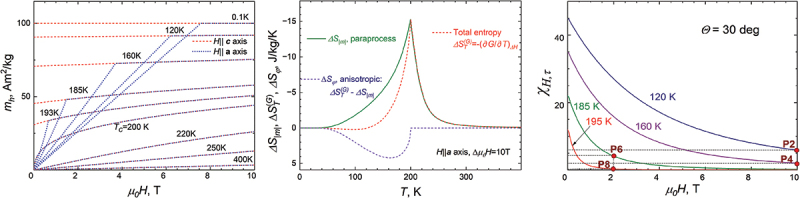

## Introduction

The magnetocaloric effect (MCE) manifests itself in alteration in the thermodynamic state of a magnetic material when it is magnetized/demagnetized in an external magnetic field *H* [1–3]. To quantify the MCE, two parameters are usually used: the adiabatic temperature change *ΔT*_*ad*_ (the magnetization process occurs with a constant total entropy of the material), and isothermal entropy change *ΔS*_*T*_ (the isothermal magnetization accompaniments by heat transfer *Q*=*ΔS*_*T*_*T* between the material and the environment) [4–6]. In contrast to intensive quantities, such as temperature or magnetic field, the entropy *S* is the extensive quantity and hence the additive function: the *S* of a macrosystem corresponds to the sum of the entropies of its constituents, which facilitates the determination of individual subsystem contributions (e.g. magnetic, structural, electronic) to the overall MCE. Through the examination of entropy changes in each subsystem separately, it is possible to accurately determine the diverse physical mechanisms underpinning this phenomenon and find strategies for maximizing the MCE [7–10].

Materials that exhibit a notable magnetocaloric effect hold promise for applications in alternative solid-state magnetic cooling technology. These magnetic refrigeration systems have the potential to operate effectively at ambient temperatures and find utility in the cryogenic liquefaction of diverse gases such as natural gas or hydrogen [11–14]. To enhance the efficiency of magnetic materials as the working body of a magnetic refrigerator, several subsystems of the magnetic solid should contribute commensurately to the overall MCE. In this connection, materials with first-order transitions, where the contributions from the magnetic and structural subsystems can be comparable in magnitude but do not always coincide in sign, are being widely studied [15–20].

Another (albeit less explored) example of magnetocaloric materials with several degrees of freedom are magnetic materials with high magneto-crystalline anisotropy and, as a consequence, with a rotational MCE [21–28]. This effect refers to the change in the thermodynamic state of the magnetic material when the external magnetic field takes an angle with the direction of ‘easy’ axis magnetization [29–36]. In this case, the total entropy change *ΔS*_*T*_ arises from two distinct contributions: (1) the conventional MCE or paraprocess *ΔS*_*|m|*_ (the magnetic field diminishes the thermal motion of magnetic moments, thereby increasing the magnetisation vector in modulus and reducing the entropy of the magnetic subsystem); (2) the rotational MCE *ΔS*_*φ*_ (the magnetization vector changes in direction, and the associated change in the anisotropy energy leads to an additional contribution to the total entropy change) [37–40].

Along with identifying the contributions from various subsystems to the overall magnetocaloric effect, another important task is to determine the mutual influence of such subsystems during the magnetization process and calculate the thermodynamic cross-coupling coefficients that describe such interaction [41–46]. A system endowed with magnetic anisotropy can be regarded as multicaloric with two generalized thermodynamic forces: magnetic field *H* and mechanical torque *τ*. Consequently, such a system provides a framework for elucidating and quantifying these cross-coupling coefficients.

We believe that it is crucial to acknowledge the substantial impact of magnetic anisotropy on the magnetocaloric effect, spanning from the vicinity of the Curie temperature to lower temperatures. Incorporating this influence is imperative for a more comprehensive understanding of magnetic phase transitions. This significance is accentuated in the current context, where magnetic cooling emerges as a promising technology, particularly for gas liquefaction, notably hydrogen. For low temperatures, the most effective materials are those with a high content of rare earth elements, and, accordingly, with potentially high magnetocrystalline anisotropy. Therefore, accounting for the anisotropic contribution and developing adequate modelling approaches are paramount when assessing the magnetocaloric effect at lower temperatures.

In this work, we used the molecular field approach [47–51], to model a single crystalline material with uniaxial magnetic anisotropy. We have shown that our model is consistent from thermodynamical point of view, and all thermodynamic quantities obtained within the model satisfy thermodynamic relations for a given system. We distinguish the contributions to the total isothermal change in entropy *ΔS*_*T*_ from (1) the entropy of the paraprocess *ΔS*_*|m|*_ (the conventional magnetocaloric effect) and (2) the entropy associated with the processes of rotation of the magnetization vector *ΔS*_*φ*_. Obtained equality Δ*S*_*T*_ = Δ*S*_*|m|*_ + Δ*S*_*φ*_ demonstrates that the model is internally consistent and applicable to anisotropic magnets as multi-calorific materials. Finally, we modelled the situation when both magnetic field *H* and torque *τ* are applied, simulating the multicaloric behaviour of the magnetically anisotropic sample, and determined the cross-coupling coefficients $\chi_{\tau ,H}={\left(\frac{\partial \tau }{\mathrm{\partial H}}\right)}_{T,\theta }$ and $\chi_{H,\tau }=-{\left(\frac{\mathrm{\partial m}}{\partial \theta }\right)}_{T,H}$ for our system. The results obtained here can be used not only for evaluation of the effectiveness of using highly anisotropic materials in magnetic cooling devices but also allow to estimate the maximum attainable rotational MCE of any given compound and, upon comparison with other multicaloric systems, can be the foundation for building a general theory of this intriguing phenomenon.

## Mean-field approach for material with uniaxial magnetic anisotropy

In general, the magnetic moment of an atom or ion arises from both spin and orbital contributions. However, in the present study, we intentionally simplified the model to enhance its usability and reduce complexity. This approach is justified by our primary objective: to study the key trends in the magnetocaloric behaviour of an anisotropic material and to explore the potential for interpreting it within the framework of multicaloric effects.

Within the framework of the molecular field model [49,51], the total energy density of the sample can be written as follows:

(1) $$E=E_{\mathrm{exch}}+E_{\mathrm{th}}+E_{H}+E_{\mathrm{an}}$$

*E*_*exch*_ is the exchange energy, or, equivalently in the framework of mean-field theory, the energy of the magnetisation vector in the molecular field $H_{\mathrm{mol}}=\mu_{0}\frac{1}{2}n_{w}\sigma m_{0}$:

(2) $$E_{\mathrm{exch}}=-\mu_{0}H_{\mathrm{mol}}\sigma m_{0}=-\mu_{0}\frac{1}{2}n_{w}\sigma^{2}m_{0}^{2}$$

Here *μ*_*0*_ is the magnetic constant, *n*_*w*_ is the molecular field parameter, *σ* is the reduced magnetisation $\sigma =\frac{m\left(T\right)}{m_{0}}$ (1≤σ≤0), *m(T)* is the magnetisation per unit volume at a certain temperature *T* and external field *H*, *m*_*0*_ is the spontaneous magnetisation at 0 K. For the spin of $\frac{1}{2},$ $m_{0}=\mu_{B}N_{v}$, where, in turn, *μ*_*В*_ is the Bohr magneton and *N*_*v*_ is the number of atoms per unit volume.

It is important to note that the molecular field parameter is related to the Curie temperature *T*_*C*_ through a well-known relation involving the Curie constant *C*: $T_{C}=Cn_{w}$, while $C=\frac{\mu_{0}\mu_{B}^{2}g^{2}N_{v}J\left(J+1\right)}{3k_{B}}$, where *k*_*B*_ is the Boltzmann constant, *g* is Landé g-factor (in our case *g* = 2), and *J* is the total angular momentum quantum number of the ion or atom (in our case *J*=*S*). In many studies, this relation is used to estimate the *n*_*w*_ from the experimentally known *T*_*C*_. In contrast, in this work, we treated the *n*_*w*_ as an input quantity and determined *T*_*C*_ as an outcome of the modelling. Notably, the resulting Curie temperature was fully consistent with the aforementioned relation.

The thermal energy *E*_*th*_ of magnetic moments with spin $\frac{1}{2}$ can be written using the configuration entropy *S*_*|m|*_ in the following way [52,53]:

(3) $$E_{\mathrm{th}}=N_{v}k_{B}TS_{\left|m\right|}=-N_{v}k_{B}T\left[\ln\left(2\right)-\frac{1}{2}\left\{\left(1-\sigma \right)\ln\left(1-\sigma \right)+\left(1+\sigma \right)\ln\left(1+\sigma \right)\right\}\right]$$

*T* is the temperature, and the expression in square brackets on the right-hand side represents the entropy of the paraprocess *S*_*|m|*_ written as a function of the reduced magnetisation σ. Subindex |m| denotes that the entropy *S*_*|m|*_ corresponds to the change of magnetization in magnitude, not in the direction.

The energy density of the material in the external field has the following form:

(4) $$E_{H}=-\mu_{0}\sigma m_{0}H\cos\left(\theta -\varphi \right)$$

where *H* is the external field, *θ* is the angle between the direction of the external magnetic field and the easy axis of magnetization, in the following termed the ***c***-axis of the crystal, and *φ* is the angle between the magnetization and the ***c***-axis.

The last term in expression (1) is the anisotropy energy:

(5) $$E_{\mathrm{an}}=K_{1}\left(T\right)\mathrm{si}n^{2}\left(\varphi \right)$$

where *K*_*1*_*(T)* is the first anisotropy constant, which depends on temperature. Since the angle *φ* between the magnetization and the ***c***-axis is an order parameter, along with the magnitude of the magnetic moment *σ*, the *φ* depends on the temperature and the applied field, however, in Eq (5) and below, for convenience, we use the notation *σ* and *φ* instead of *σ(T)* and *φ(T)*. To model the temperature dependence of *K*_*1*_ we used the classical Zener approximation [54–57], which relates the *K*_*1*_ to the temperature dependence of magnetization:

(6) $$K_{1}\left(T\right)=K_{o}\sigma^{3}\left(T\right)$$

where *K*_*0*_ is the anisotropy constant at 0 K.

It is important to note that the demagnetizing field plays a significant role in real materials, particularly under low magnetic fields where the role of magnetic domains is essential. In such cases, shape anisotropy can be exploited to achieve a substantial magnetocaloric response even in small field, comparable with the demagnetization field of the sample [58]. However, in the present study, we focused on the effects of magnetocrystalline anisotropy. To maintain a manageable level of model complexity, the influence of the demagnetizing field on magnetization rotation processes was not considered.

For all simulations carried out in this work, we used the following parameters: *K*_*0*_ = 3 × 10^6^ J/m^3^ , *n*_*w*_  = 300, $N_{v}=$ 8.49 × 10^28^, *m*_*0*_  = 787.4 kA/m. However, since the majority of publications on this topic use a specific magnetization per mass unit, for better comparison with the literature we present our result using specific magnetization *m*_*0*_ = 100 Am^2^/kg with the density of the material *ρ*  = 7874 kg/m^3^. We chose these parameters mainly because they describe a material with a Curie temperature of 200 K and a high anisotropy field of 7.6 T at 0.1 K, which, in combination with a sufficiently high magnetization, allows us to consider the phenomenon of the rotating magnetocaloric effect in all sorts of applications. Such material is similar, for example, to Fe_2_P magnetocaloric material with *T*_*C*_ = 218 K, *m*_*0*_ = 120 Am^2^/kg, *K*_*1*_(0)~2.5–2.7 × 10^6^ J/m^3^ (Ha ~6.5 T at 10 K) [59].

The total energy *E* (1) is a function of only two variables, *σ* and *φ*, so numerically minimizing the energy by varying these two order parameters, we can find the value of specific magnetization *m* and its projection on the direction of the external magnetic field $m_{h}=\left|m\right|\cos\left(\theta -\varphi \right)$ for each field and temperature. Since the entropy *S*_*|m|*_ obtained by formula (3) is a function of *σ*, and, accordingly, a function of *m*, we can construct magnetic entropy *S*_*|m|*_*(T)* and magnetic entropy change Δ*S*_*|m|*_*(H)*_*T,θ*_. If the magnetic field is applied along the easy direction of magnetization of the single crystal (in our case it is the ***c***-axis), only the paraprocess contributes to the total entropy change. However, when the field is applied along the hard axis (***a***-axis), in the fields below the anisotropy field *H*_*a*_ the magnetization rotation process occurs, and together with Δ*S*_*|m|*_ we should see additional contribution to entropy from the magnetization rotation processes Δ*S*_*φ*_, associated with the changes in the second order parameter (or degree of freedom) *φ*. It is known [32,33,60,61] that when the spontaneous magnetization vector is rotated by an angle *φ*, the associated entropy change Δ*S*_*φ*_ can be determined as

(7) $$\Delta S_{\varphi }=-\left(\frac{\partial K_{1}}{\partial T}\right)\left\{\sin^{2}\left(\varphi \right)-\sin^{2}\left(\varphi_{0}\right)\right\}$$

where $\varphi_{0}$ is the initial angle between the magnetization and ***c***-axis of the crystal. Thus, Equations (3) and (7) allow us to determine the conventional Δ*S*_*|m|*_ and anisotropic Δ*S*_*φ*_ contributions to the total magnetocaloric effect.

The value of total entropy change Δ*S*_*T*_ (which is the sum of Δ*S*_*|m|*_ and Δ*S*_*φ*_) can be found in various ways, and all of them are based on writing of the infinitesimal reversible change in the Gibbs free energy as a function of its variable *T*, *H,* and *θ* as follows:

(8) dG=−sdT−mdH+τdθ

where *τ* is mechanical torque and *θ* is the angle between the c-axis and external magnetic field. In the framework of our mean-field model, the *τ* can be calculated in two ways [62–64]:

(9) $$\tau =2K_{1}\sin\varphi \cos\varphi =K_{1}\sin2\varphi$$

(10) $$\tau =\mathrm{mH}\sin\left(\theta -\varphi \right)$$

The total entropy change Δ*S*_*T*_ can be determined as a derivative of the Gibbs energy *G* with respect to the temperature *T*:

(11) $$\Delta S_{T}^{\left(G\right)}={\left(\frac{\partial G}{\mathrm{\partial T}}\right)}_{H,\theta }$$

Additionally, for isothermal magnetization processes or isofield heating/cooling, the Maxwell’s relation is used:

(12) $$\Delta S_{T}=\overset{H_{2}}{\underset{H_{1}}{\int }}{\left(\frac{\partial \left|m\right|\cos\left(\theta -\varphi \right)}{\mathrm{\partial T}}\right)}_{H,\theta }\mathrm{dH}$$

Another Maxwell’s relation is applied if the anisotropic single crystal is rotated isothermally in the magnetic field from the initial angle *θ*_*1*_ to the final angle *θ*_*2*_.

(13) $$\Delta S_{T}=-\overset{\theta_{2}}{\underset{\theta_{1}}{\int }}{\left(\frac{\partial \tau }{\mathrm{\partial T}}\right)}_{\theta ,H}\mathrm{d\theta}$$

Furthermore, the third Maxwell’s relation allows to determine the cross-coupling coefficients *χ*_*τ,H*_ and *χ*_*H,τ*_:

(14) $$\chi_{\tau ,H}={\left(\frac{\partial \tau }{\mathrm{\partial H}}\right)}_{T,\theta }=-{\left(\frac{\mathrm{\partial m}}{\partial \theta }\right)}_{T,H}=\chi_{H,\tau }$$

## Thermodynamic consistency of multicaloric effects in magnetically anisotropic materials: disentangling contributions from paraprocess ΔS_|m|_ and rotational magnetocaloric effect ΔS_φ_ to the total magnetic entropy change ΔS_T_

**Figure 1.**
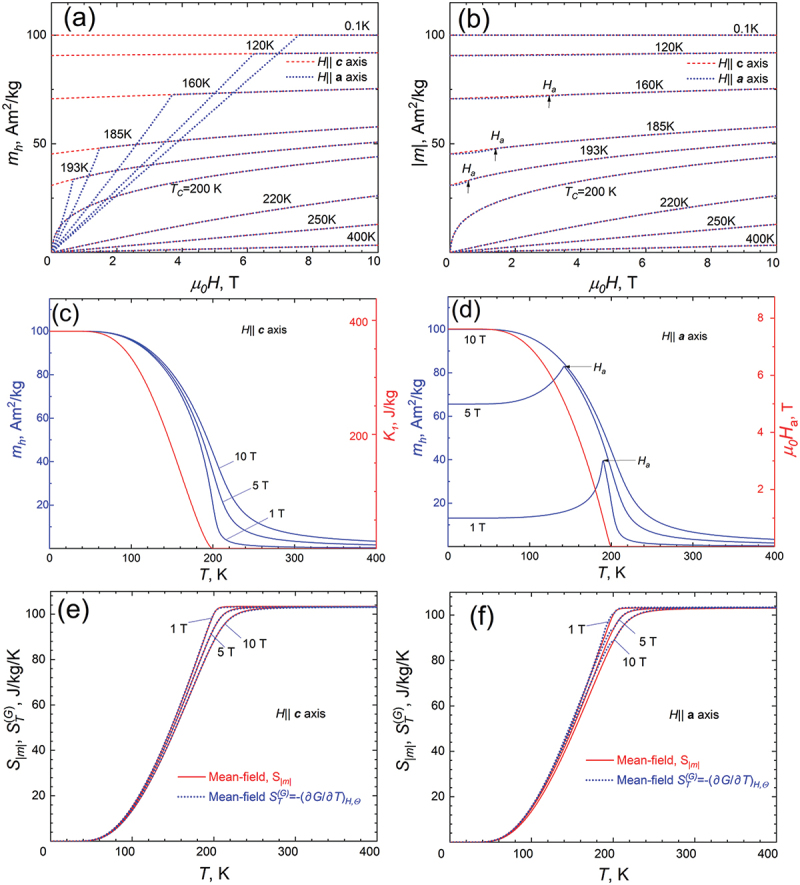
(a) Field dependencies of the projection of magnetization *m*_*h*_*(H)* on the direction of the external magnetic field, calculated along the easy (c-axis) and hard (a-axis) magnetization direction. (b) Field dependencies of spontaneous magnetization vector length *|m|(H)*, calculated for the same case as presented in fig. (a). (c) Temperature dependencies of magnetization *m*_*h*_*(T)*, the field is applied along the ***c***-axis (easy direction). The red curve (right Y-axis) shows the temperature dependence of the anisotropy constant *K*_*1*_ (Zener model). (d) *m*_*h*_*(T)*, the field is applied along ***a***-axis (hard direction). (e,f) temperature dependencies of magnetic entropy in the fields 1, 5, and 10 T, applied along easy (e) and hard (f) directions. The red curves correspond to the configurational magnetic entropy *S*_*|m|*_ (eq. (3)), while the blue curves correspond to the total entropy $S_{T}^{\left(G\right)}$ determined from the derivative of the Gibbs energy with respect to temperature.

Figure 1(a) shows the field dependences of the projection of specific magnetization *m*_*h*_*(H)*_*T*_ to the direction of magnetic field, calculated at several selected temperatures. The magnetic field is applied along the ***c***-axis (easy axis of magnetization, there is no rotational MCE) and the ***a***-axis (hard axis, paraprocess coexists with the rotation of the magnetization vector). It can be seen that after reaching the anisotropy field $H_{a}\left(T\right)=\frac{2K_{1}\left(T\right)}{m\left(T\right)}$, the *m*_*h*_ values are the same for the easy and hard directions. Below the Curie temperature (*T*_*C*_ = 200 K), for fields lower than *H*_*a*_, the *m*_*h*_*(H)*_*T*_ along the hard direction has a linear character, which confirms the correctness of the model used. Figure 1(b) shows the field dependences of the modulus of magnetization vector *|m|* for the same case shown in Figure 1(a). It is important to note that when the magnetization vector makes some angle with the direction of the external field, the *|m|* is somewhat smaller, and only in a field equal to *H*_*a*_ (shown by the arrows in Figure 1(b)) the *|m|* values are identical. Since the entropy of the paraprocess is a function of *|m|*, it is logical to expect that this contribution to the entropy should be smaller when the sample is magnetized in the hard direction.

Figure 1(c) and 1(d) show the temperature dependences of the magnetization *m_h_(T)_H_* calculated for three different fields applied in the easy (Figure 1c,d) directions. For the hard direction, the spikes in the *m_h_(T)_H_* dependences are observed at those temperatures at which the condition *H_a _*= *H* is satisfied. These figures also show the temperature dependences of the magnetic anisotropy constant *K_1_* obtained using Zener’s formula (6).

Figure 1(e,f) depict the temperature dependences of *S*_*|m|*_, calculated by using Equation (3), and $S_{T}^{\left(G\right)}$, calculated by using Equatio (11), these simulations were carried out for the same fields as in Figure 1(c,d). As can be seen from Figure 1(e,f), the configurational entropy *S*_*|m|*_ (red curves) is almost identical when the sample is magnetized in the easy and hard directions, however, small difference between *S*_*|m|*_ and $S_{T}^{\left(G\right)}$ is observed in temperatures not exceeding the Curie temperature of the sample. This difference indicates that when magnetizing along the hard axis, the magnetocaloric effect cannot be described only by configurational entropy *S*_*|m|*_, and the involvement of additional terms (contributions) in the total *S*_*T*_ is necessary.

Temperature dependencies of total entropy $S_{T}^{\left(G\right)}\left(T\right)$ are shown in Figure 1(e,f) as blue lines. When the magnetic field is applied along the easy direction (Figure 1(e)) there is no rotation of magnetization vector and the $S_{T}^{\left(G\right)}$ exactly corresponds to configurational entropy $S_{\left|m\right|}$. However, when the sample is magnetized along the hard direction, the configurational entropy and the total entropy have different values, and this discrepancy is especially clear near the Curie temperature. All this unambiguously points to the fact that if the magnetic field is applied along the hard direction, there is an additional entropy contribution *S*_*φ*_, which corresponds to the processes of rotation of the sample magnetization vector *m*.

**Figure 2.**
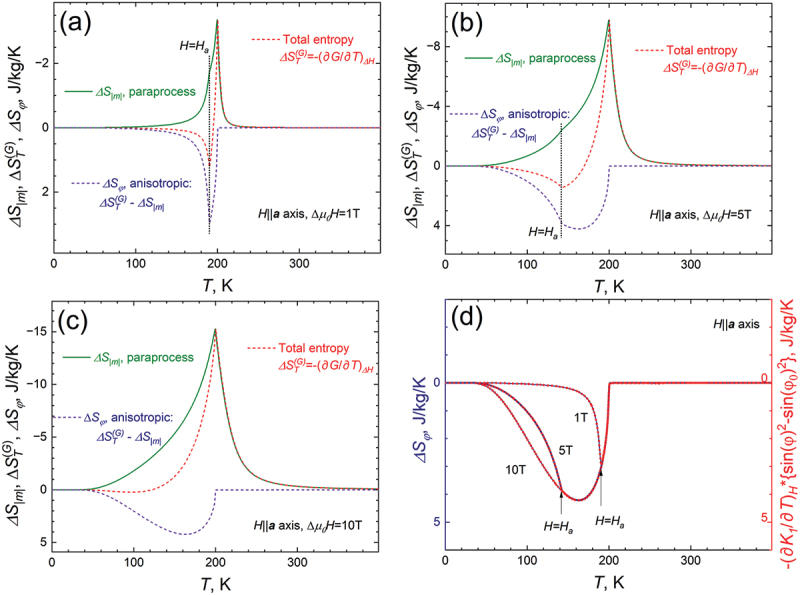
(a–c), temperature dependencies of changes in configuration entropy $\Delta S_{\left|m\right|}\left(T\right)$ (green curve), rotational entropy $\Delta S_{\varphi }\left(T\right)$ (blue curve) and total entropy $\Delta S_{T}^{\left(E\right)}\left(T\right)$ (red curve) calculated for the fields of 1 T (a), 5 T (b) and 10 T (c), *H*||***a***-axis. The $\Delta S_{\varphi }\left(T\right)$ was calculated by molecular-field approach (blue curve) and by using eq. (7) (red curve), both obtained curves are identical.

Figure 2(a,b,c) show the temperature dependencies of configurational entropy change $\Delta S_{\left|m\right|}\left(T\right)$ (Equation (3), dark green curve), the total entropy change $\Delta S_{T}^{\left(G\right)}\left(T\right)$ defined as the derivative of the total Gibbs free energy by temperature, (Eq (11), red curve), and their difference, which should represent nothing else but the contribution to the total entropy change from the magnetization rotation $\Delta S_{\varphi }\left(T\right)$. Figure 2(a) shows these dependencies for a field of 1 T and Figure 2(b) has a field of 5 T applied, both of these magnetic fields are lower than the anisotropy field of the single crystal at 0 K (*H*_*a*_(0) = 10.5 T). In Figure 2(c), the magnetic field 10 T is larger than *H*_*a*_ in the whole temperature range. If the magnetic field is not directed along the easy magnetization direction, one can see that ∆S_m_T and ∆$S_{\varphi }T$ compete with each other, leading to a decrease in the total MCE.

Since our model gives the value of the anisotropy constant *K*_*1*_ and the angle *φ* for all fields and temperatures, we can apply Equation (7) and compare the results with the rotational magnetocaloric effect determined within the molecular-field model as $\Delta S_{\varphi }=\Delta S_{T}-\Delta S_{\left|m\right|}$ and shown in Figure 2(a–c). Figure 2(d) shows that the $\Delta S_{\varphi }\left(T\right)$, obtained from the difference between $\Delta S_{T}^{\left(G\right)}\left(T\right)$ and $\Delta S_{\left|m\right|}\left(T\right)$, and the $\Delta S_{\varphi }\left(T\right)$ calculated by Equation (7), coincide completely, which confirms the correctness of using Equation (7) to determine the rotational contribution to the MCE.

**Figure 3.**
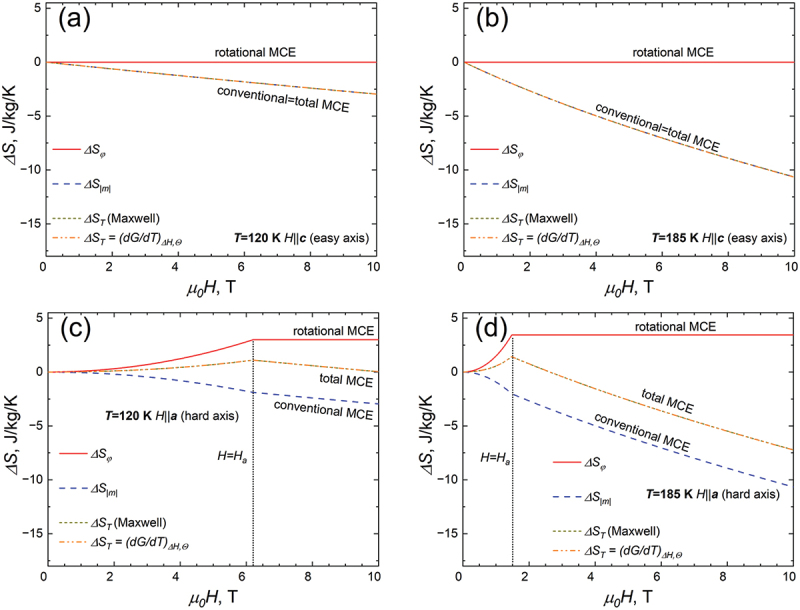
Field dependencies of various contributions to the total entropy change $\Delta S_{T}^{\left(G\right)}\left(H\right)$ for two distinct temperatures, 120 K (a,c) and 185 K (b,d). Simulations were conducted for both the easy axis (*H||**c***, (a,b)) and the hard axis (*H||**a***, (c,d)) of magnetization.

Figure 3 shows the field dependencies of different contributions to the total entropy change $\Delta S_{T}^{\left(G\right)}\left(H\right)$ obtained for two selected temperatures, (120 K, Figure 3(a,c)) and (185 K, Figure 3(b,d)). The simulations were carried out for the easy axis (*H||**c***-axis, Figure 3(a,b)) and hard axis (*H||**a***-axis, Figure 3(c,d)) of magnetization. One can see that if the magnetic field is applied along the easy direction, the MCE has only one contribution – the entropy of paraprocess $\Delta S_{\left|m\right|}\left(H\right)$, which can be found by using Eq (11) or Eq (12). When the magnetic field is applied along the hard direction of magnetization (*H||**a***-axis), both $\Delta S_{\left|m\right|}\left(H\right)$ and $\Delta S_{\varphi }\left(H\right)$ have a significant impact on the $\Delta S_{T}$ (Figure 3(c,d)). Since the conventional and rotational MCE have a different sign, the $\Delta S_{T}$ obtained along the hard direction of magnetization is significantly lower than in the case when magnetic field is applied along ***c***-axis.

## Exploring multicaloric potential of magnetically anisotropic system: sequential and simultaneous application of generalized thermodynamic forces H and τ, determination of cross-coupling coefficients χ(_H,τ_) and χ(_τ,H_). The entropy associated with the interaction of both subsystems ∆S_coupling_

**Figure 4.**
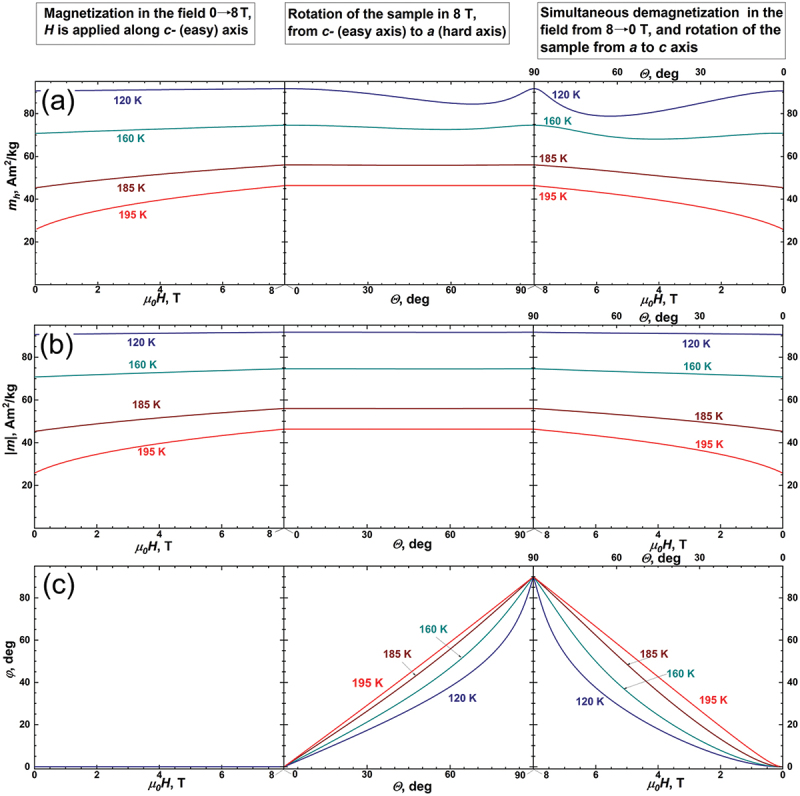
The behavior of projection of magnetization *m*_*h*_ on the external field *H* (a), spontaneous magnetization |*m|* (b) and angle *φ* which magnetization takes with the ***c***-axis during multicaloric thermodynamic cycle (c). Left column: magnetic field rises from zero to 8 T, *H||**c***-axis (easy axis); central column: the sample is rotated in the constant magnetic field of 8 T from ***c***- to ***a***-axis; right column: both thermodynamic forces are applied, magnetic fields is reducing to zero simultaneously with rotating of the sample ***a***- to ***c***-axis. The modelling was done for 120 K, 160 K, 185 K and 195 K.

The molecular field model we use in this work allows us not only to separate different contributions to the overall change in entropy $\Delta S_{T}$, but also to simulate the multicaloric effect, both for the case when several generalized thermodynamic forces (here *H* and τ) are applied sequentially to the sample, and for the case when these forces are applied simultaneously. Figure 4(a) shows the projection of magnetization *m*_*h*_ in the direction of the external magnetic field *H*, Figure 4(b) displays the absolute value of the spontaneous magnetization *|m|*, and Figure 4(c) depicts the behaviour of the angle *φ* which magnetization takes with the ***c***-axis of the crystal. The modelling was done for four selected temperatures 120 K, 160 K, 185 K, and 195 K. The left column shows the field dependencies *m*_*h*_*(H)*_*T,θ*_, *|m|(H)*_*T,θ*_ and *φ(H)*_*T,θ*_ when the magnetization process takes place along the ***c***-axis (*θ = 0*), the magnetic field changes from 0 to 8 T. The central column corresponds to the rotation of the sample from *θ* = 0 to θ = 90^°^ in the constant field of 8 T by means of torque moment *τ*, and the angular dependencies of *m*_*h*_*(θ)*_*T,H*_, *|m|(θ)*_*T,H*_ and *φ(θ)*_*T,H*_ are presented. In the right column, the sample subjected to both generalized thermodynamic forces *H* and *τ*, the sample rotates from *θ* = 90^°^ to *θ* = 0 and, at the same time, the magnetic field changes from 8 T to zero.

**Figure 5.**
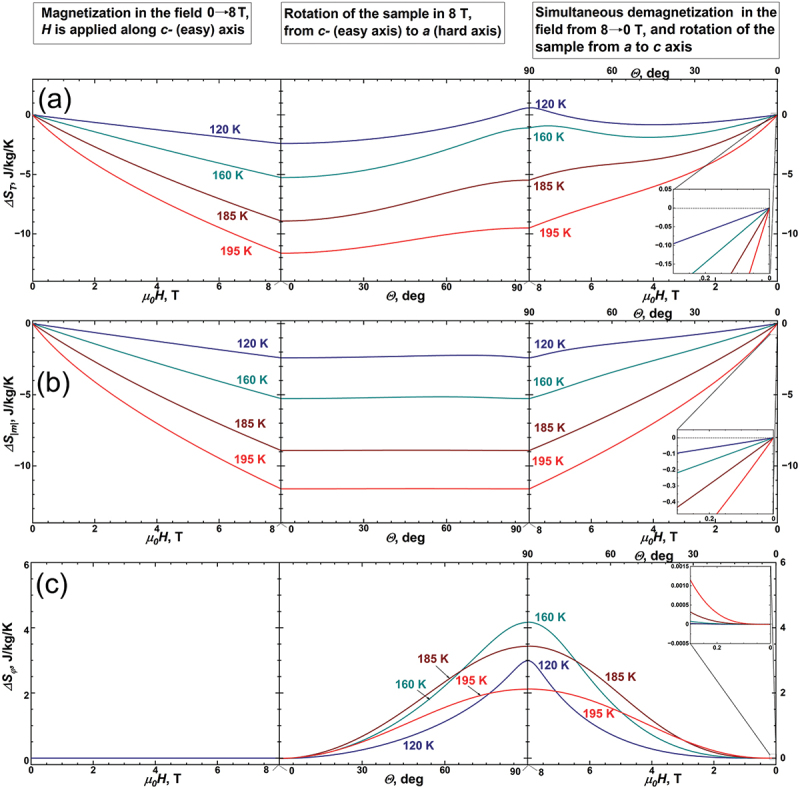
The changes in the total entropy $\Delta S_{T}$ (a), conventional entropy of paraprocess $\Delta S_{\left|m\right|}$ (b) and rotational entropy $\Delta S_{\varphi }$ (c) taking place for multicaloric cycle shown in Fig 4. The inserts on the right side show that after such a multicaloric cycle, the total, conventional and rotational entropy changes are zero.

Figure 5 shows the changes in the total entropy change $\Delta S_{T}^{\left(G\right)}$ (a), conventional entropy change of paraprocess $\Delta S_{\left|m\right|}$ (b) and rotational entropy change $\Delta S_{\varphi }$ (c), all taking place for such a multicaloric cycle. Notably, as the thermodynamic system returns to its initial state, the $S_{\left|m\right|}$, $\Delta S_{\varphi }$ and $\Delta S_{T}$ revert to zero. To illustrate this behaviour of entropy in detail, the inserts are added to the right side of Figure 5(a–c). Since our cycle is a reversible equilibrium thermodynamic cycle and entropy is a function of state, the zero entropy change reaffirms the consistency of our model.

**Figure 6.**
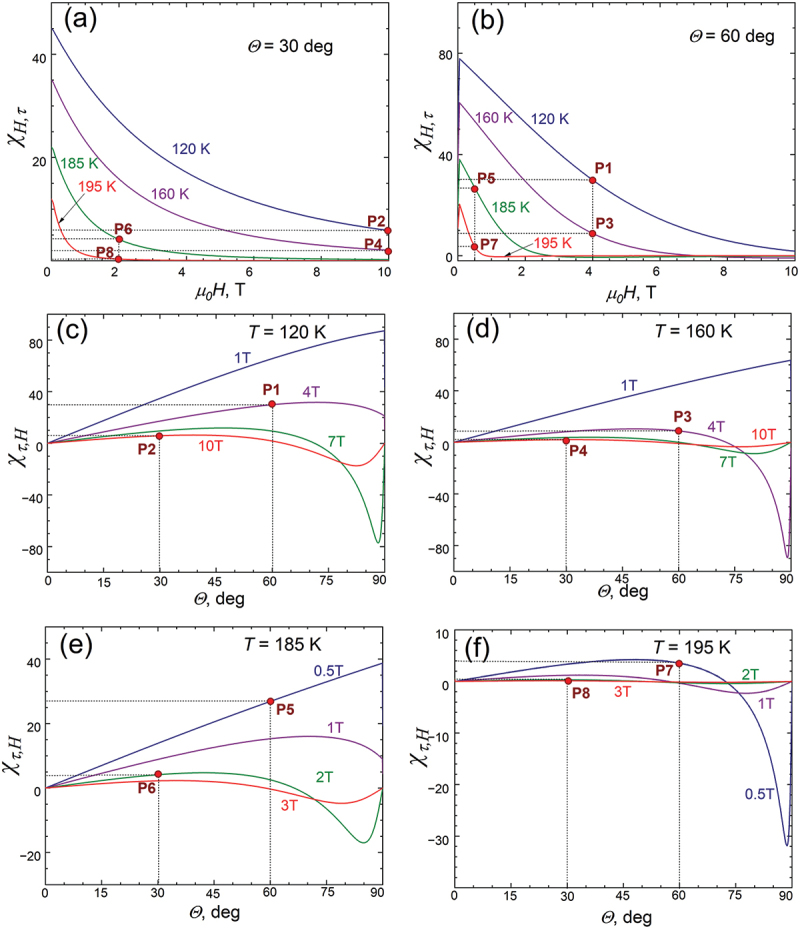
The angular dependencies of cross-coupling coefficient $\chi_{H,\tau }=-{\left(\frac{\mathrm{\partial m}}{\partial \theta }\right)}_{T,H}$ (a,b) and field dependencies of cross-coupling coefficient $\chi_{\tau ,H}={\left(\frac{\partial \tau }{\mathrm{\partial H}}\right)}_{T,\theta }$ (c,d,e,f). The simulations were performed for 4 selected temperatures of 120 K, 160 K, 185 K and 195 K. Eight points P1, P2, … ,P8 with different sets of *T*, *H* and *θ* parameters are shown with red circles. The numerical values of these eight points are given in Table 1.

Given that if the angle *φ* changes with the field (*H* makes an angle with the c-axis (*θ* ≠ 0)), or if the magnetization *m*_*h*_ changes when the sample is rotated in a constant magnetic field by means of the torque τ, we can use Equation (14) to find the cross-coupling coefficients $\chi_{\tau ,H}={\left(\frac{\partial \tau }{\mathrm{\partial H}}\right)}_{T,\theta }$ and $\chi_{H,\tau }=-{\left(\frac{\mathrm{\partial m}}{\partial \theta }\right)}_{T,H}$ that links the changes in *τ* or *m* to the changes in non-conjugated thermodynamic forces, respectively, angle *θ* and field *H*, which, in turn, can quantitatively determine the mutual intertwisting of the conventional and rotational MCE. By definition, these two coefficients must be equal for the same values of *T*, *H*, and *θ*. Figure 6(a,b) shows the field dependences of the $\chi_{H,\tau }$ for *θ*  = 30° and *θ* = 60°, respectively, the magnetic field was varied from 0 to 10 T, and calculations were performed for 4 selected temperatures of 120 K, 160 K, 185 K and 195 K. Figure 6(c–f) depicts the angular dependencies of the $\chi_{\tau ,H}$ calculated for the same selected temperatures. Eight points P1, P2, … ,P8 with different sets of *T*, *H* and *θ* variables were selected (they are shown in Figure 6 with red circles) and the values of both $\chi_{H,\tau }$ and $\chi_{\tau ,H}$ cross-coupling coefficients are summarized in Table 1. It can be seen that for all selected combinations of *T*, *H* and *θ*, $\chi_{H,\tau }={\left(\frac{\partial }{\partial H}{\left(\frac{\mathrm{\partial G}}{\partial \theta }\right)}_{T,H}\right)}_{T,\theta }={\left(\frac{\partial }{\partial \theta }{\left(\frac{\mathrm{\partial G}}{\mathrm{\partial H}}\right)}_{T,\theta }\right)}_{T,H}=\chi_{\tau ,H}$. The numerical values of these eight points with the same set of *T*, *H* and *θ* are given in Table 1. It can be seen that for all selected combinations of *T*, *H* and *θ*, the thermodynamic identity $\chi_{H,\tau }=\chi_{\tau ,H}$ is satisfied.

**Table 1.** $\chi_{H,\tau }$and $\chi_{\tau ,H}$ cross-coupling coefficients obtained in 8 different points (see fig 6) along with the *T/T*_*C*_ and *H/H*_*a*_ ratios for each point.

point	P1	P2	P3	P4	P5	P6	P7	P8
*T* *H* *θ*	120K, 4T, 60°	120K, 10T, 30°	160K, 4T, 60°	160K, 10T, 30°	185K, 0.5T 60°	185K, 2T 30°	195K, 0.5T 60°	195K, 2T 30°
$\chi_{H,\tau }$	29.9	5.8	8.8	8.8	26.9	4.1	3.6	3.6
$\chi_{\tau ,H}$	29.9	5.8	8.8	8.8	26.9	4.1	3.6	3.6
T/T_c_	0.6	0.6	0.8	0.8	0.925	0.925	0.975	0.975
H/H_a_	0.526	1.315	0.526	1.315	0.065	0.263	0.065	0.263

**Figure 7.**
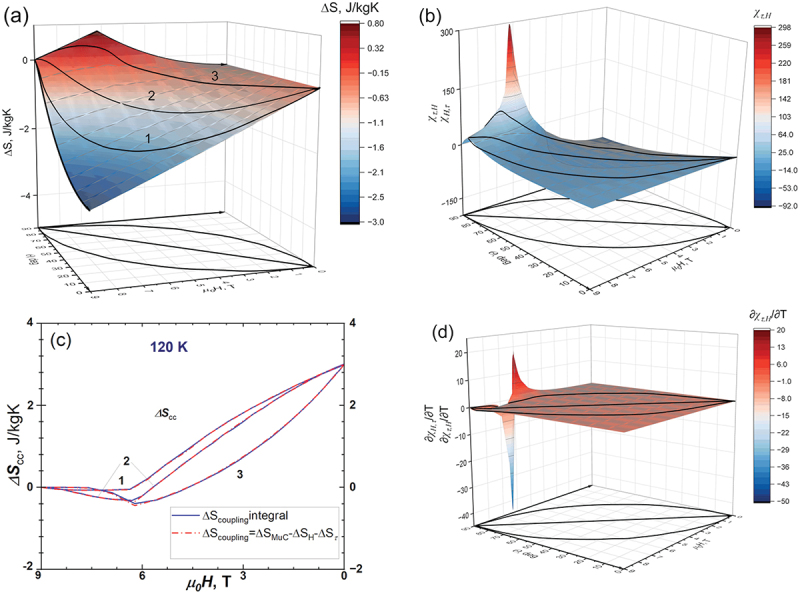
The changes in the total entropy $\Delta S_{T}$ (a), the cross-susceptibilities $\chi_{H,\tau }$ and $\chi_{\tau ,H}$(b), comparison of $\;\Delta S_{\mathrm{coupling}}$, calculated by mean field theory (red) and by eq. (16) (blue) (c) and the cross-susceptibility $\chi_{H,\tau }$ derivative by temperature (d).

Multiferroic materials, due to the strong coupling between different degrees of freedom, tend to exhibit multicaloric effects caused by the application or removal of different external fields or forces. In our case, this effect arises as a synergistic response to the combined action of magnetic field *H* and mechanical torque *τ*. Figure 7(a) shows the total entropy change for the initial state defined as *H* = 9 T, *θ* = 90° when both forces *H* and *τ* are applied simultaneously. Since entropy is a function of state, the entropy change at any point on the obtained $\Delta S_{T}\left(H,\theta \right)$ surface is independent of the particular path taken to reach that point. There are 3 selected paths in the Figure 7(a), showing as a black lines. Figure 7(b) depicted the cross-coupling coefficients $\chi_{H,\tau }$ and $\chi_{\tau ,H}$, whereas 7(c) their derivatives with respect to the temperature $\frac{\partial \chi_{H,\tau }}{\mathrm{\partial T}}$ and $\frac{\partial \chi_{\tau ,H}}{\mathrm{\partial T}}$.

It was shown in the literature [9,42–44,46] that the total multicaloric effect can be expressed as the sum of MCE obtained by application of respective field/force separately (in our case demagnetisation from 9 T to 0 T keeping the angle θ = 90° and the rotation from *θ* = 90° to *θ* = 0° keeping the field 9 T) minus the magnetic entropy change associated with cross-coupling of the subsystems of the solid $\;\Delta S_{T}=\Delta S_{T,\tau }^{\left(H\right)}+\Delta S_{T,H}^{\left(\theta \right)}-\;\Delta S_{\mathrm{coupling}}$. In turn, this allows us to calculate the change in entropy associated with the interaction of both subsystems $\;\Delta S_{\mathrm{coupling}}$ in two ways: The first way is that we can use (i) the magnetic entropy $\Delta S_{T,\tau }^{\left(H\right)}$ calculated within the framework of molecular field theory for demagnetisation in the field under constant *θ*, (ii) rotation in the constant field $\Delta S_{T,H}^{\left(\theta \right)}$ and the total magnetic entropy change $\;\Delta S_{T}$.

(15) $$\;\Delta S_{\mathrm{coupling}}=\;\Delta S_{T}-\Delta S_{T,H}^{\left(\theta \right)}-S_{T,\tau }^{\left(H\right)}$$

In the second method, we can use the cross-coupling coefficients, as it was suggested in [9,42]

(16) $$\;\Delta S_{\mathrm{coupling}}=\overset{0}{\underset{90}{\int }}\overset{0}{\underset{9}{\int }}\frac{\partial \chi_{H,\tau }\left(T,H{\;}^{\prime },\theta {\;}^{\prime }\right)}{\partial T}\mathrm{dH}{\;}^{\prime }\mathrm{d\theta}{\;}^{\prime }$$

Figure 7(d) shows the calculated $\;\Delta S_{\mathrm{coupling}}$ using Equation 15 (blue line) and Equation (16) (red lines). Both methods agree very well with each other. Moreover, this correspondence clearly indicates that a material with magnetic anisotropy is a multi-caloric material with respect to the application of magnetic field *H* (the conjugate variable is the magnetization *m*) and mechanical torque *τ* (the conjugate variable is the angle *θ* between the magnetization and *c*-axes of the crystal).

## Discussion and conclusions

Summarizing the results obtained in this work, we would like to emphasize several important points:

We have shown that a material with magnetic anisotropy can be considered as a model object for studying the multicaloric effect. Indeed, the thermodynamic state of such an object can be changed either by applying the magnetic field (generalized thermodynamic force *H*) or by mechanical rotation of the sample in the magnetic field by means of a torque τ. Thus, along with already actively studied systems with pairs of thermodynamic generalized forces, such as pressure – magnetic field, uniaxial mechanical stress – magnetic field, electric field – magnetic field, etc., we can add to our consideration a new class of objects, where the concept of the multicaloric effect is realized through a pair of generalized forces: τ - *H*.

**Figure 8.**
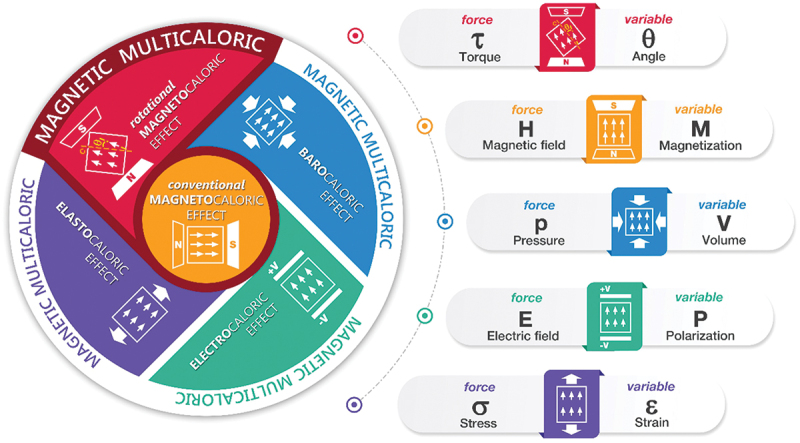
Schematic illustration of four magnetic multicaloric effects. Each effect incorporates the conventional magnetocaloric effect (center of the left diagram). The multicaloric effects shown – electrocaloric, elastocaloric, barocaloric, and the rotational magnetocaloric effect discussed in this work – are all coupled with magnetic ordering. The corresponding conjugate thermodynamic variables for each effect are indicated on the right side of the figure.

Figure 8 shows the schematic illustration of four magnetic multicaloric effects: electrocaloric, elastocaloric, barocaloric, and the rotational magnetocaloric effect discussed in this work. All these effects coupled with magnetic ordering, thus each multicaloric effect incorporates the conventional magnetocaloric effect (center of the left diagram). The corresponding conjugate thermodynamic variables for each effect are indicated on the right side of the figure.

The use of the molecular field model allows us to unambiguously separate the contributions to the total magnetocaloric effect from different subsystems of a magnetic solid (in our case we separated contribution from conventional $\Delta S_{\left|m\right|}$ and rotational $\Delta S_{\varphi }$ to the total magnetic entropy change $\Delta S_{T}$). It is important to note that due to different signs of $\Delta S_{\left|m\right|}$ and $\Delta S_{\varphi }$, in polycrystals with strong uniaxial magnetic anisotropy, the magnetocaloric effect cannot reach its optimal value.

It is noteworthy that when the sample is magnetized along the hard axis, the magnitude of the magnetization vector *|m|* for magnetic fields below *H*_*a*_ remains lower than when single crystal magnetized along the easy axis. This indicates that the rotation of magnetization and the change in the absolute value of magnetization *|m|* are interdependent processes. It is possible that a more detailed consideration of this issue within the framework of a specially extended molecular field model will help to better understand the consideration of such a phenomenon as anisotropy of magnetization [65–67].

In our model, if we change the sign of the anisotropy constant to the opposite, the ***a***-axis will become the easy axis, and ***c***-axis turns to the hard axis. In this case, the conventional (or configurational) MCE will be observed along the ***a***-axis, and both rotational and conventional MCE will be observed along the ***c***-axis. In this case, the sign of the rotational effect will not change, it will remain negative. On the other hand, the sign of the rotational effect depends on the sign of the derivative of the anisotropy constant with respect to temperature. In the case of spin-reorientation transitions, when the anisotropy constant can increase with increasing temperature, the sign of the rotational magnetocaloric effect will be positive, as was experimentally shown in our previous works [32,35].

Within the framework of the molecular field model, we have determined cross-coupling coefficients $\chi_{\tau ,H}={\left(\frac{\partial \tau }{\mathrm{\partial H}}\right)}_{T,\theta }$ and $\chi_{H,\tau }=-{\left(\frac{\mathrm{\partial m}}{\partial \theta }\right)}_{T,H}$ for anisotropic magnetic material and have shown that these coefficients satisfy the basic thermodynamic identities, for example, the equality of the second partial derivatives of the total differential of the Gibbs energy. We also confirmed that the total multicaloric effect if the material with magnetic anisotropy can be expressed as the sum of MCE obtained by application of respective field/force separately minus the magnetic entropy change associated with cross-coupling of the subsystems of the solid $\;\Delta S_{T}=\Delta S_{T,\tau }^{\left(H\right)}+\Delta S_{T,H}^{\left(\theta \right)}-\;\Delta S_{\mathrm{coupling}}$.

In conclusion, it is crucial to acknowledge the substantial impact of magnetic anisotropy on the magnetocaloric effect, spanning from the vicinity of the Curie temperature to lower temperatures. Incorporating this influence is imperative for a more comprehensive understanding of magnetic phase transitions. This significance is accentuated in the current context, where magnetic cooling emerges as a promising technology, particularly for gas liquefaction, notably hydrogen. For low temperatures, the most effective materials are those with a high content of rare earth elements, and, accordingly, with potentially high magnetocrystalline anisotropy. Therefore, accounting for the anisotropic contribution and developing adequate modelling approaches are paramount when assessing the magnetocaloric effect at lower temperatures. Finally, extensive studies of MCE anisotropy can facilitate finding new, constructive solutions in developing magnetic refrigerators [29].

## References

[1] Weiss P, Piccard A. Le phénomène magnétocalorique. J Phys Théorique Appliquée. 1917;7(1):103–14. doi: 10.1051/jphystap:019170070010300

[2] Gschneidner A, Pecharsky VK, Tsokol AO. Recent developments in magnetocaloric materials. Rep Prog Phys. 2005;68(6):1479–1539. doi: 10.1088/0034-4885/68/6/R04

[3] Gutfleisch O, Willard MA, Brück E, et al. Magnetic materials and devices for the 21st century: stronger, lighter, and more energy efficient. Adv Mater. 2011;23:821–842. doi: 10.1002/adma.201002180 21294168

[4] Smith A, Bahl CRH, Bjørk R, et al. Materials challenges for high performance magnetocaloric refrigeration devices. Adv Energy Mater. 2012;2(11):1288–1318. doi: 10.1002/aenm.201200167

[5] Sandeman KG. Magnetocaloric materials: the search for new systems. Scr Mater. 2012;67(6):566–571. doi: 10.1016/j.scriptamat.2012.02.045

[6] Moya X, Kar-Narayan S, Mathur ND. Caloric materials near ferroic phase transitions. Nat Mater. 2014;13(5):439–450. doi: 10.1038/nmat3951 24751772

[7] Liu J, Gottschall T, Skokov KP, et al. Giant magnetocaloric effect driven by structural transitions. Nat Mater. 2012;11(7):620–626. doi: 10.1038/nmat3334 22635044

[8] Waske A, Dutta B, Teichert N, et al. Coupling phenomena in magnetocaloric materials. Energy Technol. 2018;6(8):1429–1447. doi: 10.1002/ente.201800163

[9] Stern-Taulats E, Castán T, Mañosa L, et al. Multicaloric materials and effects. MRS Bull. 2018;43(4):295–299. doi: 10.1557/mrs.2018.72

[10] Mendive Tapia E, Patrick CE, Hickel T, et al. Quantification of electronic and magnetoelastic mechanisms of first-order magnetic phase transitions from first principles: application to caloric effects in La(Fe_x_Si_1−x_)_13_. J Phys Energy. 2023;5(3):034004. doi: 10.1088/2515-7655/acd027

[11] Giauque WF. A thermodynamic treatment of certain magnetic effects. A proposed method of producing temperatures considerably below 1° absolute. J Am Chem Soc. 1927;49:1864–1870. doi: 10.1021/ja01407a003

[12] Brown GV. Magnetic heat pumping near room temperature. J Appl Phys. 1976;47(8):3673–3680. doi: 10.1063/1.323176

[13] Mezaal NA, Osintsev KV, Zhirgalova TB. Review of magnetic refrigeration system as alternative to conventional refrigeration system. IOP Conf Ser Earth Environ Sci. 2017;87:032024. doi: 10.1088/1755-1315/87/3/032024

[14] Steven Brown J, Domanski PA. Review of alternative cooling technologies. Appl Therm Eng. 2014;64(1–2):252–262. doi: 10.1016/j.applthermaleng.2013.12.014

[15] Gràcia-Condal A, Stern-Taulats E, Planes A, et al. Caloric response of Fe49Rh51 subjected to uniaxial load and magnetic field. Phys Rev Mater. 2018;2(8):084413. doi: 10.1103/PhysRevMaterials.2.084413

[16] Gottschall T, Gràcia-Condal A, Fries M, et al. A multicaloric cooling cycle that exploits thermal hysteresis. Nat Mater. 2018;17(10):929–934. doi: 10.1038/s41563-018-0166-6 30202111

[17] Scheibel F, Gottschall T, Taubel A, et al. Hysteresis design of magnetocaloric materials—from basic mechanisms to applications. Energy Technol. 2018;6(8):1397–1428. doi: 10.1002/ente.201800264

[18] Gutfleisch O, Gottschall T, Fries M, et al. Mastering hysteresis in magnetocaloric materials. Philos Trans R Soc A Math Phys Eng Sci. 2016;374(2074):20150308. doi: 10.1098/rsta.2015.0308 PMC4938067 27402928

[19] Gruner ME, Keune W, Landers J, et al. Moment-volume coupling in La(Fe1−xSix)13. Phys Status Solidi Basic Res. 2018;255(2):1700465. doi: 10.1002/pssb.201700465

[20] Davarpanah A, Belo JH, Amaral VS, et al. On the optimization of magneto-volume coupling for practical applied field magnetic refrigeration. Phys Status Solidi Basic Res. 2019;256(3):1–6. doi: 10.1002/pssb.201800419

[21] de Sousa VSR, Plaza EJR, Reis MS, et al. Investigation on the magnetocaloric effect in DyNi_2_, DyAl_2_ and Tb1−nGdnAl_2_ (n=0, 0.4, 0.6) compounds. J Magn Magn Mater. 2009;321(20):3462–3465. doi: 10.1016/j.jmmm.2009.06.054

[22] Jin J-L, Zhang X-Q, Li G-K, et al. Giant anisotropy of magnetocaloric effect in TbMnO3 single crystals. Phys Rev B. 2011;83(18):184431. doi: 10.1103/PhysRevB.83.184431

[23] Maraytta N, Voigt J, Salazar Mejía C, et al. Anisotropy of the magnetocaloric effect: example of Mn_5_Ge_3_. J Appl Phys. 2020;128(10):103903. doi: 10.1063/5.0020780

[24] Liu Y, Petrovic C. Anisotropic magnetocaloric effect and critical behavior in CrCl_3_. Phys Rev B. 2020;102(1):014424. doi: 10.1103/PhysRevB.102.014424

[25] Wang K, Zhang M, Liu J, et al. Crystal structure, spin reorientation, and rotating magnetocaloric properties of NdCo_5-x_Si_x_ compounds. J Appl Phys. 2019;125(24):125. doi: 10.1063/1.5093708

[26] Zhao X, Zheng X, Luo X, et al. Large magnetocaloric effect and magnetoresistance in ErNi single crystal. J Mater Sci Technol. 2021;86:56–63. doi: 10.1016/j.jmst.2020.12.072

[27] Zhao X, Zheng X, Qi J, et al. Anisotropic magnetocaloric effect and magnetoresistance in antiferromagnetic HoNiGe_3_ single crystal. Intermetallics. 2021;138:107307. doi: 10.1016/j.intermet.2021.107307

[28] Nikitin SA, Ivanova TI, Zvonov AI, et al. Magnetization, magnetic anisotropy and magnetocaloric effect of the Tb_0.2_Gd_0.8_ single crystal in high magnetic fields up to 14 T in region of a phase transition. Acta Mater. 2018;161:331–337. doi: 10.1016/j.actamat.2018.09.017

[29] Kuz’min MD, Tishin AM. Magnetic refrigerants for the 4.2–20 K region: garnets or perovskites? J Phys D Appl Phys. 1991;24(11):2039–2044. doi: 10.1088/0022-3727/24/11/020

[30] Lima AL, Gschneidner KA, Pecharsky VK. Anisotropic materials: a way to increase the efficiency of magnetic refrigeration. J Appl Phys. 2004;96(4):2164–2168. doi: 10.1063/1.1767969

[31] Nikitin SA, Tishin AM, Leontiev PI. Magnetocaloric effect and pressure influence on dysprosium single crystal magnetization in the range of magnetic phase transition. J Magn Magn Mater. 1991;92(3):405–416. doi: 10.1016/0304-8853(91)90855-5

[32] Nikitin SA, Skokov KP, Koshkid’ko YS, et al. Giant rotating magnetocaloric effect in the region of spin-reorientation transition in the NdCo_5_ single crystal. Phys Rev Lett. 2010;105:137205. doi: 10.1103/PhysRevLett.105.137205 21230806

[33] Basso V, Sasso CP, Küpferling M, et al. Er_2_Fe_14_B single crystal as magnetic refrigerant at the spin reorientation transition. J Appl Phys. 2011;109(8):083910. doi: 10.1063/1.3567925

[34] Fries M, Skokov KP, Karpenkov DY, et al. The influence of magnetocrystalline anisotropy on the magnetocaloric effect: a case study on Co_2_B. Appl Phys Lett. 2016;109(23):232406. doi: 10.1063/1.4971839

[35] Skokov KP, Pastushenkov YG, Nikitin SA, et al. Rotational magnetocaloric effect in the Er_2_Fe_14_B single crystal. IEEE Trans Magn. 2016;52(5):1–4. doi: 10.1109/TMAG.2016.2530138

[36] Zhang J, Hu Y. Role of magnetocrystalline anisotropy on anisotropic magnetocaloric effect in single crystals. Appl Phys Lett. 2021;119(21):119. doi: 10.1063/5.0068818

[37] de Oliveira NA, von Ranke PJ. Theoretical aspects of the magnetocaloric effect. Phys Rep. 2010;489(4–5):89–159. doi: 10.1016/j.physrep.2009.12.006

[38] Balli M, Roberge B, Fournier P, et al. Review of the magnetocaloric effect in RMnO_3_ and RMn_2_O_5_ multiferroic crystals. Crystals. 2017;7(2):44. doi: 10.3390/cryst7020044

[39] Konieczny P, Czernia D, Kajiwara T. Rotating magnetocaloric effect in highly anisotropic Tb_III_ and Dy_III_ single molecular magnets. Sci Rep. 2022;12(1):16601. doi: 10.1038/s41598-022-20893-2 36198759 PMC9534846

[40] Bae JH, Cho KK, Lee JW, et al. Magnetic entropy changes for the rotating magnetocaloric effect in RB_4_ (R = Gd, Tb, Dy, Ho, Er, and Tm). J Magn Magn Mater. 2023;576:170767. doi: 10.1016/j.jmmm.2023.170767

[41] Meng H, Li B, Ren W, et al. Coupled caloric effects in multiferroics. Phys Lett A. 2013;377(7):567–571. doi: 10.1016/j.physleta.2012.12.033

[42] Planes A, Castán T, Saxena A. Thermodynamics of multicaloric effects in multiferroics. Philos Mag. 2014;94(17):1893–1908. doi: 10.1080/14786435.2014.899438 PMC4938063 27402925

[43] Liang F-X, Hao J-Z, Shen F-R, et al. Experimental study on coupled caloric effect driven by dual fields in metamagnetic heusler alloy Ni_50_Mn_35_In_15_. APL Mater. 2019;7(5):1–8. doi: 10.1063/1.5090599

[44] Liu Y, Xu Z, Qiao K, et al. Strengthened caloric effect in MnCoSi under combined applications of magnetic field and hydrostatic pressure. J Mater Sci. 2021;56(36):20060–20070. doi: 10.1007/s10853-021-06546-1

[45] Gràcia-Condal A, Planes A, Mañosa L, et al. Magnetic and structural entropy contributions to the multicaloric effects in Ni-Mn-Ga-Cu. Phys Rev Mater. 2022;6(8):084403. doi: 10.1103/PhysRevMaterials.6.084403

[46] Hao J-Z, Wang B, Hu F-X, et al. Competitive driving effect on calorics by dual-fields in ferroic materials with strong magnetostructural coupling. Acta Mater. 2024;265:119596. doi: 10.1016/j.actamat.2023.119596

[47] Bean CP, Rodbell DS. Magnetic disorder as a first-order phase transformation. Phys Rev. 1962;126(1):104–115. doi: 10.1103/PhysRev.126.104

[48] Kato T, Nagai K, Aisaka T. A model of magneto-structural phase transition in MnAs. J Phys C Solid State Phys. 1983;16(16):3183–3196. doi: 10.1088/0022-3719/16/16/020

[49] Vives E, Castán T, Planes A. Unified mean-field study of ferro- and antiferromagnetic behavior of the ising model with external field. Am J Phys. 1997;65(9):907–913. doi: 10.1119/1.18681

[50] Moreno-Ramírez LM, Franco V. Reversibility of the magnetocaloric effect in the Bean-Rodbell model. Magnetochemistry. 2021;7(5):60. doi: 10.3390/magnetochemistry7050060

[51] Piazzi M, Bennati C, Curcio C, et al. Modeling specific heat and entropy change in La(Fe–Mn–Si)_13_–H compounds. J Magn Magn Mater. 2016;400:349–355. doi: 10.1016/j.jmmm.2015.07.055

[52] Smart JS. Magnetic structure transitions. Phys Rev. 1953;90(1):55–58. doi: 10.1103/PhysRev.90.55

[53] Basso V, Piazzi M, Bennati C, et al. Hysteresis and phase transition kinetics in magnetocaloric materials. Phys Status Solidi. 2018;255(2):1700278. doi: 10.1002/pssb.201700278

[54] Zener C. Classical theory of the temperature dependence of magnetic anisotropy energy. Phys Rev. 1954;96(5):1335–1337. doi: 10.1103/PhysRev.96.1335

[55] Keffer F. Temperature dependence of ferromagnetic anisotropy in cubic crystals. Phys Rev. 1955;100(6):1692–1698. doi: 10.1103/PhysRev.100.1692

[56] Carr WJ. Temperature dependence of ferromagnetic anisotropy. J Appl Phys. 1958;29(3):436–437. doi: 10.1063/1.1723171

[57] Callen HB, Callen E. The present status of the temperature dependence of magnetocrystalline anisotropy, and the l(l+1)/2 power law. J Phys Chem Solids. 1966;27(8):1271–1285. doi: 10.1016/0022-3697(66)90012-6

[58] Almeida R, Freitas SC, Fernandes CR, et al. Rotating magnetocaloric effect in polycrystals—harnessing the demagnetizing effect. J Phys Energy. 2024;6(1):015020. doi: 10.1088/2515-7655/ad1c61

[59] Caron L, Hudl M, Höglin V, et al. Magnetocrystalline anisotropy and the magnetocaloric effect in Fe2P. Phys Rev B. 2013;88(9):094440. doi: 10.1103/PhysRevB.88.094440

[60] Akulov NS, Kirensky LW. Über einen neuen magnetokalorischen effekt. J Phys. 1940;3:31–34.

[61] Ivanovskii VI. Magnetocaloric effect in the region of rotation fields. Phys Met Met. 1959;7:24.

[62] Burd J, Huq M, Lee EW. The determination of magnetic anisotropy constants from torque curves. J Magn Magn Mater. 1977;5(2):135–141. doi: 10.1016/0304-8853(77)90179-2

[63] Ono F, Yamada O. Temperature dependence of the uniaxial magneto-crystalline anisotropy energy of Co. J Phys Soc Jpn. 1979;46(2):462–467. doi: 10.1143/JPSJ.46.462

[64] Wang X, Wu R, Wang D, et al. Torque method for the theoretical determination of magnetocrystalline anisotropy. Phys Rev B. 1996;54(1):61–64. doi: 10.1103/PhysRevB.54.61 9984224

[65] Bolyachkin AS, Neznakhin DS, Bartashevich MI. The effect of magnetization anisotropy and paramagnetic susceptibility on the magnetization process. J Appl Phys. 2015;118(21):18–21. doi: 10.1063/1.4936604

[66] Gignoux D, Givord F, Lemaire R. Magnetic properties of single crystals of GdCo2, HoNi_2_, and HoCo_2_. Phys Rev B. 1975;12(9):3878–3884. doi: 10.1103/PhysRevB.12.3878

[67] Kuz’min MD, Skokov KP, Radulov I, et al. Magnetic anisotropy of La_2_Co_7_. J Appl Phys. 2015;118(5):053905. doi: 10.1063/1.4927849

